# Coping with online racism: Patterns of online social support seeking and anti-racism advocacy associated with online racism, and correlates of ethnic-racial socialization, perceived health, and alcohol use severity

**DOI:** 10.1371/journal.pone.0278763

**Published:** 2022-12-02

**Authors:** Brian TaeHyuk Keum, Xu Li

**Affiliations:** 1 Department of Social Welfare, University of California, Los Angeles, Los Angeles, California, United States of America; 2 Department of Educational Psychology, University of Wisconsin-Milwaukee, Milwaukee, Wisconsin, United States of America; The Hong Kong Polytechnic University, HONG KONG

## Abstract

Given the emerging public health concerns of online racism, we examined potential coping approaches for racial/ethnic minority adults. Using a latent class regression model (*N =* 407), we examined patterns of online social support seeking and anti-racism advocacy engagements that were associated with online racism among racial/ethnic minority adults. We also examined whether these patterns were associated with ethnic-racial socialization messages (cultural socialization, promotion of mistrust, preparation for racial bias), perceived health, and alcohol use severity. Three distinct latent groups were identified with meaningful group differences: triggered/reactive (alcohol use risk, higher promotion of mistrust), moderate engagement (no risk), disengaged/non-reactive (higher promotion of mistrust, higher cultural socialization, alcohol use risk) groups. Online social support seeking and advocacy engagement may have both benefits and costs in coping with online racism. Those who engage at optimal/balanced levels appear to report better well-being. Implications for research and practice are discussed.

## Introduction

Racism (categorization based on phenotypic characteristics or ethnic/ancestral group affiliation) results in trauma at multiple levels [1], including individual (e.g., social exclusion, violence), cultural (e.g., White supremacy and cultural devaluation of people of color), and systemic inequities (e.g., policies and structures that systematically disadvantage people of color). Racism thrives pervasively and explicitly on the internet [2, 3]. Mechanisms such as online anonymity have allowed individuals to openly share their racist ideologies without feeling accountable, inhibited, and needing to be politically correct [3]. Research suggests that racial victimization in online settings is significantly linked to mental health costs (e.g., distress) among adolescents and emerging adults of color [2, 4].

Despite emerging public health concerns of online racism [4, 5] little research has examined the potential mechanisms that promote coping strategies in dealing with online racism, particularly for racial/ethnic minority adults. Extant research suggests that conducive racism coping strategies have involved social support seeking, resistance, and advocacy that seem to be buffers of poorer health and engagement in risky self-medication strategies such as alcohol use [6]. Furthermore, the way people cope with racism has been found to depend on ethnic-racial socialization (E-RS), a process through which individuals learn from their parents and guardians about race and ways to deal with racism starting as early as their critical childhood periods [7]. Yet, these factors have not been examined alongside online racism. Thus, using a latent class regression model, (1) we explored racial/ethnic minority adults’ distinct patterns of online social support seeking and anti-racism advocacy in response to online racism, and (2) whether the patterns may be differentially associated with type and salience of E-RS messages received across a lifetime and varying levels of health risk (perceived health, alcohol use severity).

### Coping with online racism: Online social support and anti-racism advocacy

Within various risk and resiliency frameworks [8], racism has been well-established as major developmental adversity and s risk factor that exacerbates a host of psychosocial and health costs among racial/ethnic minority individuals. With the persistent, explicit, and pervasive presence of racism on the internet in today’s digitally-driven social dynamics, online racism has added greater weight to such risks. Recent operationalization of online racism [2] suggests that people may experience online racism through individual racial cyber-aggression (e.g., receiving racist messages), vicariously witnessing racial cyber-aggression (e.g., seeing others receive racist comments), and by consuming online content that conveys the reality, presence, and violence of systemic racism in society (e.g., information on various systemic disparities against racial minority individuals, online media dehumanizing entire cultures of racial/ethnic minority groups, physically violent police brutality videos toward Black individuals). Compared to offline racism, online racism accounted for unique variance in racial/ethnic minority adults’ psychological distress, and perceptual influences such as unjust views of society, and stress about future experiences [2] suggesting that online racism may be a unique contemporary risk factor.

The prevalence of online racist content is likely an unjust digital burden on racial/ethnic minority individuals and even facilitates traumatic exposure to contents of racism and racial violence on the internet [3, 4]. For example, frequent exposure to viral online videos documenting police brutality on Black individuals has been associated with symptoms of trauma [9, 10]. This is supported by the racial trauma theory [11], which suggests that racism-related stressors are experienced chronically and additively over time and can induce a sense of ongoing trauma. Carter [11] notes that this is particularly the case if an individual perceives certain racism-related stressors to be outside of their ability to cope. In such cases, racial/ethnic minority individuals may engage in social adaptions such as hypervigilance, social avoidance, numbing, and emotional dysregulation that is ultimately insidious and maladaptive. These approaches may appear seemingly adaptive to reduce the harmful costs of racial discrimination, but in doing so yields comorbid consequences in psychological (e.g., distress, loneliness), and behavioral (e.g., alcohol use; [12, 13]) domains that in turn, increase susceptibility to psychopathology [11]. For instance, Wei and colleagues [14] found that some racial/ethnic minority adults choose to cope with racism via detachment (disengaging from problem-solving and social support), internalization (self-blame), and drug and alcohol use. In particular, studies have found that online racism was significantly linked to significant increases in alcohol use severity among racial minority emerging adults with greater risk among women [12, 13]. Maladaptive coping strategies such as alcohol use have been linked to poorer mental health such as greater depressive symptoms, and lower self-esteem [14, 15].

The racial trauma theory [11] also suggests that individuals may engage in adaptive coping in response to online racism. Extant literature on racism coping provides a significant foundation for examining potential processes for adaptive coping with online racism. In general, racism coping strategies have involved social support seeking, resistance, and advocacy [6]. Of note, seeking social support from allies or finding counterspaces that affirm and validate racial injustices appeared to be the most common strategy [6]. These counterspaces [16] likely allow victims of racism to seek instrumental (e.g., learning microinterventions to counter racist messages from others) and emotional support (e.g., validation of anger and sadness), and collaboratively develop strategies for more active and resistant racism coping approaches [17].

Similarly, seeking social support in online spaces may be a potent strategy for dealing with online racism. Given the convenience of finding like-minded people on the Internet, people can seek and find support from various groups promoting racial equality and social justice [2, 3]. These online social spaces may provide a safe and empowering environment for people to share their experiences of racism and receive validation and empathy from others. The “virtual courage” stemming from online anonymity may also work in favor of sharing social justice commitments and making a public anti-racist stance even for those who may not have had the courage and motivation to do so in the offline context [18]. Indeed, recent sociological studies have found that racial/ethnic minority individuals seek social support on online counterspaces to deal with racial discrimination on online social platforms [19–21].

### Coping with online racism and ethnic-racial socialization

Developmental theories on risk and protective frameworks [8] suggest that racial/ethnic minority individuals may employ more adaptive coping strategies than maladaptive approaches based on the presence of certain protective factors during their developmental periods that promote greater resilience among some racial/ethnic minority adults compared to those who lacked such protective factors. Protective factors may benefit individuals to strengthen their ability to cope with racism-related stressors so that they are less likely to experience symptoms or maladaptive coping related to racial trauma [11]. Notably, one widely studied protective factor is Ethnic-Racial Socialization (E-RS), or parents’ messages to their children on issues of race and racism, that was primarily developed from studies of Black/African American parents [22]. Theories such as the life course perspective [23] and the social cognitive learning theory [24] suggest E-RS as an important developmental parental influence on their children’s racial beliefs and attitudes through modeling and verbal/non-verbal communication [25]. Although there are racial group differences in socialization [7], racial/ethnic minority parents generally convey measurable patterns of messages about race and racism to their children [26]. Hughes and colleagues [26] found that parents communicate racial messages to their children in the following ways: (a) *cultural socialization*; messages on racial and cultural pride, (b) *preparation for bias*; or messages acknowledging racial discrimination and promoting ways to deal with it, and (c) *promotion of mistrust*; messages of caution and mistrust in others who might perpetuate racial discrimination. Given the distinct typologies and valence of these messages, E-RS dimensions have been found to have mixed roles as a buffer in mitigating the negative impact of racism [7].

Individuals who grew up receiving racially affirming cultural socialization messages (e.g., messages that promote pride, strength, and beauty of own race and cultural roots) are likely to cope with online racism through positive, resilient, self-affirming strategies. For instance, research on adolescents of color’s experience suggests that positive ethnic identity and affirmation buffer the relationship between online racism and psychological costs [5]. Given the focus on racially affirming messages, cultural socialization has been consistently found as a buffer between racism and its links to psychological distress and substance use [7]. In particular, a study [27] found that cultural socialization decreased substance use via stronger social bonds. Hence, it is likely that those who grew up socialized with messages of racial and cultural pride may be more socially resilient in seeking online social support and advocating for racial justice [28] in response to online racism and engage in less risky coping strategies such as alcohol use.

On the other hand, there may be a greater likelihood of engaging in risky and internalizing coping strategies for racial/ethnic minority adults who grew up predominantly socialized with messages preparing them for racial bias and promoting mistrust of others. Preparation for bias was only a significant buffer between racial discrimination and lower self-esteem for African American youth but not a significant buffer between racism and poorer well-being for Asian American [29] and Latinx college students [30]. For individuals who have been socialized to adopt mistrust and avoidance of others to cope with racism, the literature suggests that it actually exacerbates greater psychological deficits. In contrast to cultural socialization findings, research [27] found that promotion of mistrust was linked to lower social bonds, which in turn was associated with greater substance use. Interestingly, Thai and colleagues [29] found evidence of promotion of mistrust as a buffer between racial microaggressions and lower self-esteem for Asian Americans. The nuanced racial group differences suggest potential context- and racial identity-dependent mechanisms of these messages. For example, Su and colleagues [31] found that messages promoting mistrust may only be detrimental when received from friends and not parental figures for African American young adults. In general, however, it seems likely that racial/ethnic minority adults socialized through mistrust and racial bias preparation messages navigate the race relations with the burden of socially avoidant and racism-related vigilance perspectives. These individuals may be less likely to engage in online social support seeking and advocacy in response to online racism and be at greater risk for detached, self-induced risky coping such as alcohol use [2, 3].

### The present study

Based on the review, we conducted latent class regression to explore distinct patterns of online social support seeking and anti-racism advocacy that may be associated with online racism among racial minority adults. First, we examined whether greater exposure to online racism would be significantly associated with greater online social support seeking and anti-racism advocacy. Based on this model, we then examined whether there may be distinct latent classes that represent varying levels of engagement (e.g., more, less, not engaged) in online social support seeking and anti-racism advocacy. We did not anticipate a specific number of classes but hypothesized that a particular group may be more engaged while another group may be less engaged or avoidant in online social support seeking and anti-racism advocacy, and that there may also be a distinct group that only engages in one and not the other (e.g., only support seeking). Furthermore, given prior evidence on online racism’s impact on poorer mental health and increased alcohol use severity [2, 3], we examined whether perceived health, alcohol use severity, and the three dimensions of E-RS were significantly associated with each of the classes as correlates. Based on the literature, we hypothesized that a class representing greater online social support seeking and anti-racism advocacy would be associated with more positive perceived health, low alcohol use severity, high cultural socialization, and low promotion of mistrust and preparation for bias. We hypothesized that the reverse would be true for a class representing low or no engagement in online social support seeking and anti-racism advocacy. Finally, we explored race by gender group membership probabilities of each class given our review on the nuanced racial differences in racial socialization experiences.

Additionally, given the different social dynamics in the offline and online environments [3], we expected that our predicted patterns may not align with major trends in the extant literature that has focused mostly on offline racism. Although the literature on online experiences is limited, research [19] found that online counterspaces likely have coping benefits for dealing with online racial victimization while another study [32] found that men of color engaged in emotional desensitization and silence as a way to deal with racism in online gaming. To and colleagues [21] found that majority of the participants did not trust public online social forums as spaces for support seeking regarding racism. Furthermore, while engaging in anti-racism advocacy may be self-affirming, it may also be an emotionally-charged and exhausting process [33]. Thus, we anticipated our analyses to potentially yield classes that may be counterintuitive to current literature but align with findings on online social dynamics.

## Materials and methods

### Participants

A total of 407 racial/ethnic minority adults (*M*age = 34.12; *SD* = 11.19; range = 18–63) participated in the study. About 57% (*n* = 232) of the participants identified as women, 41% (*n* = 167) as men, and 2% (*n* = 8) as transgender. Approximately 40% (*n* = 163) of the participants identified as Black/African American, 23% (*n* = 94) as Asian/Asian American, 20% (*n* = 81) as Hispanic/Latinx American, 9% (*n* = 37) as Multiracial, 5% (*n* = 20) as Native American, 2% (*n* = 8) as Middle Eastern, and 1% (*n* = 4) as Native Hawaiian/Pacific Islander. About 84% (*n* = 342) of the participants identified as heterosexual, 9% (*n* = 37) as bisexual, 2% (*n* = 8) as lesbian, 2% (*n* = 8) as gay, 2% (*n* = 8) as asexual, and 1% (*n* = 4) as queer. About 38% (*n* = 155) of the participants had a four-year college degree, 21% (*n* = 85) had some college (no degree), 15% (*n* = 61) had a two-year college degree, 12% (*n* = 49) had a master’s degree, 9% (*n* = 37) had a high school diploma, 3% (*n* = 12) had a professional degree (e.g., MD, JD), 1% (*n* = 4) had a doctoral degree (e.g., Ph.D.), and 1% (*n* = 4) had less than a high school diploma.

### Measures

#### Perceived online racism

The Perceived Online Racism Scale (PORS) was used to assess people’s experiences of racist online interaction and exposure to racist online content and information [2]. The 30 items of the PORS span three domains: personal experience of racial cyber-aggression (e.g., “I have received racist insults regarding my online profile [e.g., profile pictures, user ID.]”), vicarious exposure to racial cyber-aggression (e.g., “I have seen other racial/minority users being treated like a second-class citizen.”), and online-mediated exposure to racist reality (e.g., “I have been informed about a viral/trending racist event happening elsewhere [e.g., in a different location].” “Seen online videos (e.g., YouTube) that portray my racial/ethnic group negatively,” “Been informed about unfairness in healthcare for racial/ethnic minority indivdiuals [e.g., biased quality of treatment, insurance issues]). Responses are rated on a five-point Likert-type scale ranging from 1 (*Never*) to 5 (*All the time*), and higher scores indicated greater exposure to online racism. Keum and Miller [2] established good initial psychometric properties for the PORS with good Internal consistency estimates (.90 to .95 across the subscales), construct validity relationships with racism-relate stress, psychological distress, and unjust views of society, and measurement invariance across race (Black, Asian, Latinx, Multiracial) and gender (men and women). The Cronbach’s alphas for the total scale score was .96 for the current study.

#### Alcohol use severity

The 10-item Alcohol Use Disorders Identification Test (AUDIT; [34]) was used to assess participants’ alcohol use severity. The AUDIT items represent alcohol consumption (items 1–3), drinking behavior/dependence (items 4–7), and alcohol-related problems or consequences (items 8–10), and is scored by summing up all of the items. The first eight items are scored on a 5-point Likert-type scale ranging from 0 to 4, and the last two are scored on 3-point Likert-type scale with values of 0, 2, and 4. The total scores range from 0 to 40, with higher scores indicating more severe alcohol problems. The AUDIT cutoff score for harmful use of alcohol is generally recommended as 8, although for women a lower cutoff score of 5 or 6 is suggested [35]. The mean AUDIT score in our sample was 6.66 (SD = 7.96; Range 0–34), with nearly 30% (n = 121) endorsing harmful use of alcohol (score ≥ 8). The AUDIT has shown measurement invariance in racially diverse university student sample [36]. The total score Cronbach’s alpha for the current study was .93.

#### Ethnic-racial socialization

Ethnic-racial socialization from parents was assessed using the adapted version of the 16-item Hughes and Johnson [37] socialization scale. This multidimensional scale includes 5 items for cultural socialization (e.g., “Talked to you about important people or events in your group’s history”), 8 items for preparation for bias (e.g., “Talk to you about others trying to limit you because of race”), 3 items for the promotion of mistrust (e.g., “Done or said things to keep you from trusting kids of other races”). Participants rated how frequently they heard these messages throughout their lifetime from 1 *(never)* to 5 *(often*). Higher scores indicate more racial socialization messages from their parents on each subscale. Validity has been demonstrated through its correlations with self-esteem, ethnic identity, and racial discrimination [26, 37] and the measure has yielded good reliability with Black, Asian, and Latinx groups [29–31]. Internal consistency estimates for the three scales were adequate for the current study; cultural socialization, α = .88; preparation for bias, α = .93; promotion of mistrust, α = .87.

*Anti-racism advocacy*. The 21-item Anti-Racism Behavioral Inventory (ARBI; [38]) was used to assess participants’ anti-racism advocacy engagements. The ARBI includes the Individual Advocacy (9 items; “I interrupt racist conversations and jokes when I hear them in my family”), Institutional Advocacy (5 items; “I write letters to local and state politicians to voice my concerns about racism”), and Awareness of Racism subscales (7 items; “The police unfairly target Black men and Latinos”). Participants rated the items on a five-point Likert-type scale (*strongly disagree* to *strongly agree*), with higher scores indicating greater advocacy. ARBI was negatively related to color-blind racial attitudes and positively with the receptivity to cultural diversity [38] and showed adequate internal consistency estimate (.86) in a diverse racial/ethnic sample [39]. The Cronbach’s alpha for the total score in this study was .89.

#### Perceived health

In balancing the length of the study survey, we used a single item perceived health (“Would you say your health is excellent, very good, good, fair, or poor?”) that has been widely used in nationally representative studies with diverse racial/ethnic individuals [40]. Participants rated their responses five-point Likert-type scale (*1-poor* to *5-excellent*). The single item has been shown to have good predictability on health-related outcomes such as mortality rates and disability [40].

#### Online social support seeking

Given that no measure exists on seeking social support and counterspace to deal with online racism, we developed the Coping Online with Racism Scale (CORS). Item development was guided by the qualitative themes on coping and anti-racism advocacy [18] and theoretically guided by relevant dimensions in prior adaptations of Carver’s COPE inventory [41] to online contexts [42]. Participants were asked to respond to the items about their efforts to deal with online racism on the internet on a five-point Likert-type scale (1-*never* to 5-*very often*). Ten items were developed based on the following aspects: Active Coping (“I block or unfollow people that do not share my goal on abolishing racism.”), Planning (“I look for online resources.”), Emotional Support (“I seek emotional support in online groups/social media.”), Instrumental Support (“I follow or connect with people who helps me to cope.”), and Venting of Emotions (“I let out my frustration or anger in online groups/social media.”). The Cronbach’s alpha for the current sample was .94. Please see the results section for initial psychometric evidence and the S1 Appendix for a full list of items.

### Procedure

Ethics approval for the study was granted by the Institutional Review Board at the University of Maryland (1431058–1). The study was determined exempt from full review according to federal regulations. Online informed consent was provided and obtained from all participants. Informed consent information was provided on the first page of the online survey (hosted on Qualtrics). After reading the consent, participants were only allowed to proceed with the study survey if they click a button that indicate that they agree to the consent and would like to proceed. Participants were recruited via convenience sampling by advertising study invitation messages in online groups on social media (e.g., Facebook, Reddit) with significant traffic of racial/ethnic minority individuals. The survey was advertised as an assessment of participants’ online racial/ethnic experiences. The survey consisted of study variable measures and demographic items. The inclusion criteria for the study were: (1) 18 years old or older, (2) self-identify as a racial/ethnic minority, and (3) currently reside in the United States. The survey took 15 to 20 minutes to complete and included two attention check items (e.g., “Please choose always”). Participants were given the option to sign up for a raffle to win one of two $50 Amazon gift cards.

## Results

### Preliminary analysis

A total of 922 participants opened the survey. Among them, 234 cases were removed for not meeting the inclusion criteria, 78 were removed for failing the attention check items, and 203 were removed for missing more than 20% of the data. Little’s missing completely at random (MCAR) test was significant, suggesting that missing data in our sample were not completely random, χ2 (280) = 365.105, *p* <. 001. The response rate was 44%. Missing data was handled using full information maximum likelihood estimation. Descriptive statistics and bivariate correlations are listed in Table 1.

**Table 1:** Descriptive statistics and bivariate correlations of study variables.

	Descriptives						Correlation						
Variables	Min	Max	*M*	*SD*	Skewness	Kurtosis	1	2	3	4	5	6	7
1. PORS	1.00	5.00	2.25	.78	.45	-.28							
2. CORS	1.00	5.00	2.27	.96	.25	-.90	.69**						
3. ARBI	1.00	4.71	2.83	.75	-.17	-.29	.54**	.63**					
4. PB	1.00	5.00	2.64	.95	.10	-.52	.64**	.43**	.40**				
5. PM	1.00	5.00	2.27	.99	.42	-.47	.65**	.49**	.35**	.59**			
6. CS	1.00	5.00	2.68	.99	.10	-.62	.54**	.42**	.45**	.76**	.47**		
7. AUDIT	8.57	49.00	18.37	8.32	1.15	.34	.45**	.43**	.30**	.21**	.35**	.19**	
8. Health	1.00	5.00	3.45	.91	-.16	-.38	.05	-.01	.09	.02	.07	.02	.06

*Note*. PORS = Perceived Online Racism Scale; CORS = Coping Online with Racism Scale; ARBI = Anti-Racism Behavioral Inventory; PB = Preparation for Bias; PM = Promotion of Mistrust; CS = Cultural Socialization; AUDIT = Alcohol Use Severity.

*p< .05

**p< .01

***p< .001.

#### Psychometric properties of the coping online with racism scale

We randomly assigned half of our sample for exploratory factor analysis (EFA; *n* = 194) and the other half for confirmatory factor analysis (CFA; *n* = 213). Even though we hypothesized the items to load onto a single factor, we conducted an EFA with oblique Promax rotation for data-driven assessment of the factor structure of the CORS. The scree plot (sharp bend at the factor 2 mark) and parallel analysis suggested a unidimensional structure based on the observed eigenvalues greater than the random 95th percentile [43]. Based on 1000 random data sets, only the first factor had a raw data eigenvalue (6.194, 0.918, …) that was greater than the simulated random eigenvalues (1.480, 1.333, …). Bartlett’s test of sphericity was χ^2^(45) = 1434.76, *p* < .001, and the Kaiser-Meyer-Olkin measure of sampling adequacy was .90, indicating that the data were sufficiently factorable. Factor loadings of the unidimensional model were all significant and ranged from .57 to .85, with factor determinacy value of .97, and communality values ranging from .53 to .70. The CORS accounted for 58% of the variance.

We cross-validated the unidimensional model by conducting a CFA. We evaluated model fit using the following fit indices [44]: (a) comparative fit index (CFI; > .95 for good fit; .92 to .94 for adequate fit), (b) the standardized root mean square residual (SRMR; close to < .08 for acceptable fit), (c) and the root mean square error of approximation (RMSEA; close to < .08 for acceptable fit). We employed maximum likelihood estimation with standard errors and chi-square test statistic that are robust to non-normality. The CFA suggested that the unidimensional model had a good fit to the data, CFA = .970, TLI = .957, RMSEA = .07 [.046, .094], SRMR = .033. Factor loadings were significant and ranged from .71 to .89. Given overall evidence for a unidimensional model, we used the total score of CORS in our analyses.

### Main analysis

#### Model set-up

Traditionally, Latent Class Analysis (LCA) was used to identify latent clusters based on a set of categorical or continuous indicator variables (in the case of continuous indicators, it was also named Latent Profile Analysis). However, later statistical developments have advanced the LCA framework to latent variable mixture models [45, 46], which allows for the exploration of latent classes not only based on mean levels or odd ratios of the indicator variables, but also the associations between variables. The latter scenario is often called the regression mixture model, or latent class regression model, and has been used in existing research in the field of counseling/clinical psychology (e.g., [47]). In the regression mixture model, the researchers can specify both mean values of the indicator variables as well as the associations between these variables to be different across a set of latent classes. Therefore, these identified latent classes can potentially unveil clusters of individuals who not only differ on the mean levels but also the association patterns between indicator variables.

In our analysis, we selected participants’ ratings on their online racism (PORS), coping online with racism scale (CORS), and anti-racism advocacy (ARBI) to be the three indicator variables. Further, we set up the regression part of our model by regressing CORS and ARBI on PORS, which quantified how much participants’ help-seeking coping and anti-racism advocacy changed in response to one unit increase in their perceived online racism. In specifying the latent classes of the model, we allowed the mean level of PORS to be estimated freely across the latent classes, which manifested the potential different levels of perceived online racism across the identified latent classes. We also allowed the intercepts of CORS and ARBI to vary across the latent classes, which represented the “baseline” levels of participants’ help-seeking coping and anti-racism advocacy in the absence of perceived online racism (this is what the intercepts meant in the regression). Lastly, we specified the two regression paths between PORS and CORS, and between PORS and ARBI to vary. This specification could help reveal the potentially different patterns of responding to perceived online racism across the identified latent classes: did participants in different latent classes show an increase, or decrease, or no change, in their help-seeking coping and advocacy in response to one unit increase of their perceived online racism.

The regression mixture models [45] also permit researchers to include covariates that are hypothesized to be associated with the latent classes. Using a three-step approach [45], a latent class model will be estimated first based on the specified indicator variables, and then the obtained latent class membership variable together with its classification probabilities will be used to estimate how the latent class relates to the auxiliary covariates. In this study, we modeled five covariates: three racial socialization scores, perceived health, and alcohol use. Specifically, we examined whether the identified latent classes were related to participants’ racial socialization scores on preparation for bias (PB), promotion of mistrust (PM), and cultural socialization (CS) subscales, and to their scores on alcohol use (AUDIT) and perceived health.

We used Mplus 8.0 [48] to test the aforementioned model. To employ the mixture modeling technique, we used the “TYPE = MIXTURE” command and included the five covariates as auxiliary variables. To handle the nonconformity to multivariate normality, we employed the robust maximum likelihood estimator (MLR).

#### Determining the number of latent classes

To determine the number of latent classes for the model specified in Step 1, using best practices [49], we tested a series of models with increasing numbers of latent classes. Statistical scholars argued that, the most appropriate model should have small Akaike Information Criteria (AIC) estimates and Bayesian Information Criteria (BIC) estimates; in addition, all generated classes should have appropriate class sizes (more than 10% of the total sample size) so that they represent meaningful classes that are not composed of outlier cases [49]. We started with 2 classes and gradually increased to 5 classes. The overall model fit information is presented in Table 2. Both AIC and BIC indices decreased when increasing the class number from 2 to 3, which suggested improved model fit. When further increasing class numbers from 3 to 4, the AIC index decreased but the BIC index increased. The BIC index increased further when the latent class number increased from 4 to 5. The bootstrapped likelihood ratio test (BLRT; [50]) indicated that there was a significant improvement in model fit when the number of latent classes increased from 2 to 3 (2×*ΔLL* = 50.57, Δ*df* = 6, bootstrapped approximate *p* < .001), but not from 3 to 4 (2×*ΔLL* = 24.80, Δ*df* = 6, bootstrapped approximate *p* = .308). Taken together, because the 3-class solution had (a) the smallest BIC value, (b) appropriately representative class sizes (i.e., no small-sized classes), and (c) significant improvement in model fit compared to the 2-class solution and nonsignificant change compared to the 4-class solution, we determined that the 3-class solution was the best fit to the data, which was retained for further analysis.

**Table 2:** Overall model fit indices for different number of latent classes and latent class membership probabilities for the three-class solution.

Number of Latent Classes	Log Likelihood	AIC	BIC
2	-1221.78	2473.55	2533.68
3	-1196.49	2434.99	2519.17
4	-1184.09	2422.18	2530.42
5	-1173.72	2413.43	2545.73
**Most Likely Latent Class Membership**	**Average Latent Class Probabilities**		
	**Triggered/ Reactive**	**Moderate Engagement**	**Disengaged/ Non-reactive**
**Triggered/Reactive**	.76	.06	.18
**Moderate Engagement**	.12	.87	.00
**Disengaged/Non-reactive**	.21	.01	.78

*Note*. AIC = Akaike Information Criteria; BIC = Bayesian Information Criteria.

#### Inspecting the specific latent classes

Next, we proceeded to inspect the 3 latent classes that emerged from the previous step. Table 3 presents the means and the associations between the three indicator variables for each of the three latent classes, together with the difference significance tests of the means and association strengths among the three classes. Class 1 (*n* = 177, 43.5%) was characterized by a high level of perceived online racism and low levels of baseline help-seeking coping and anti-racism advocacy. Further, online racism significantly and positively predicted help-seeking coping and anti-racism advocacy in Class 1 and the prediction effects were significantly larger than those of Class 2 and 3. Class 2 (*n* = 159, 39.1%) was characterized by a low level of perceived online racism, a moderate level of baseline help-seeking coping, and a low level of baseline anti-racism advocacy. Moreover, online racism also significantly and positively predicted help-seeking coping and anti-racism advocacy in Class 2 but the prediction effects were more in the moderate range. Lastly, Class 3 (*n* = 71, 17.4%) was characterized by a high level of perceived online racism and high levels of baseline help-seeking coping and anti-racism advocacy. However, online racism did not significantly predict help-seeking coping or anti-racism advocacy in Class 3, suggesting that though Class 3 individuals showed high levels of engagement in help-seeking coping and anti-racism advocacy, these engagement behaviors were not in direct response to increased perception of online racism. Given these results, we named Class 1 the Triggered/Reactive group, Class 2 the Moderate Engagement group, and Class 3 the Disengaged/Non-reactive group.

**Table 3:** Description of the final three-class solution: Means of and associations between indicator variables.

	Triggered/ Reactive	Moderate Engagement	Disengaged/ Non-reactive	Significant Differences
	(*n* = 177)	(*n* = 159)	(*n* = 71)	
**Means**				
**PORS**	2.52***	1.77***	2.57***	C1 > C2, C3 > C2
**CORS**	.38	.94***	3.33***	C3 > C2 > C1
**ARBI**	1.85***	1.70***	2.78***	C3 > C1, C3 > C2
**Associations**				
**PORS→CORS**	.93***	.21**	-.09	C1 > C2 > C3
**PORS→ARBI**	.50***	.37**	.13	C1 > C3

*Note*. PORS = Perceived Online Racism Scale; CORS = Coping Online with Racism Scale; ARBI = Anti-Racism Behavioral Inventory. C1 = Triggered/Reactive; C2 = Moderate Engagement; C3 = Disengaged/Non-reactive. The last column (Significant Differences) compares the means and association strengths among the three latent classes and displays only significant results (any paired comparison not mentioned in this column is not significant). For example, the first row shows PORS mean score comparison results: both Triggered/Reactive and Disengaged/Non-reactive groups showed larger mean PORS scores than the Moderate Engagement group; no significant differences were found between Triggered/Reactive and Disengaged/Non-reactive groups.

*p< .05

**p< .01

***p< .001.

#### Latent classes relating to E-RS dimensions, alcohol use severity, and perceived health

Next, we examined the association between the obtained three latent classes to the five auxiliary covariates [45, 49], including the three racial socialization dimensions, alcohol use and perceived health scores. Specifically, we investigated how the levels of the five auxiliary covariates predicted the likelihood of an individual being categorized into one of the three latent classes. Estimation results are presented in Table 4. Regarding E-RS dimensions, model results demonstrated that the promotion of mistrust score was significantly associated with log odds of being in the Triggered/Reactive group in reference to the Moderate Engagement group (*b* = .92, *SE* = .34, *p* = .007; odds ratio = 2.51), and being in the Disengaged/Non-reactive group in reference to the Moderate Engagement group (*b* = .90, *SE* = .27, *p* = .001; odds ratio = 2.45). This suggested that one unit score higher in promotion of mistrust was associated with 151% higher likelihood to be in the Triggered/Reactive group, and 145% higher likelihood to be in the Disengaged/Non-reactive group, in reference to being in the Moderate Engagement group. Further, the racial pride scores were significantly associated with the log odds of being in the Disengaged/Non-reactive group in reference to the Moderate Engagement group (*b* = .65, *SE* = .31, *p* = .036; odds ratio = 1.92), suggesting that one unit score higher in racial pride was associated with 92% higher likelihood to be in the Disengaged/Non-reactive group in reference to the Moderate Engagement group. No other significant associations between E-RS scores and latent class membership were found.

**Table 4:** Prediction of latent class membership by racial socialization dimensions and health indicators.

Variables	Unstandardized B	*SE*	*p*	Odds Ratio
**Triggered/Reactive VS. Moderate Engagement (reference)**				
**PB**	.35	.36	.321	1.43
**PM**	.92	.34	.007	2.51
**CS**	.47	.32	.145	1.60
**AUDIT**	.13	.04	.000	1.14
**Health**	-.01	.23	.959	.99
**Disengaged/Non-reactive VS. Moderate Engagement (reference)**				
**PB**	-.16	.36	.660	.85
**PM**	.90	.27	.001	2.45
**CS**	.65	.31	.036	1.92
**AUDIT**	.12	.03	.001	1.12
**Health**	-.20	.20	.326	.82
**Disengaged/Non-reactive VS. Triggered/Reactive (reference)**				
**PB**	-.51	.52	.323	.50
**PM**	-.02	.27	.928	.98
**CS**	.18	.43	.673	1.20
**AUDIT**	-.02	.03	.480	.98
**Health**	-.18	.22	.409	.83

*Note*. PB = Preparation for Bias; PM = Promotion of Mistrust; CS =

Cultural Socialization; AUDIT = Alcohol Use Severity; The values indicate

likelihood to fall in a particular group in comparison to the reference group.

Compared to the Moderate Engagement group, Alcohol use was found to significantly relate to the log odds of being in the Triggered/Reactive group (*b* = .13, *SE* = .04, *p* < .001; odds ratio = 1.14), and being in the Disengaged/Non-reactive group (*b* = .12, *SE* = .03, *p* = .001; odds ratio = 1.12). This indicated that one unit score higher in alcohol use was related to a 14% higher likelihood to be in the Triggered/Reactive group and a 12% higher likelihood to be in the Disengaged/Non-reactive group, in reference to being in the moderate engagement group. Perceived health scores were unrelated to latent class membership.

#### Latent classes relating to gender by race subgroups

We conducted an exploratory analysis on the association between the three obtained latent classes and the participants’ racial and gender groups, with the attempt to provide a depiction of each of the three classes. Due to sample size limitations, we included only participants who reported gender identity as cisgender women or cisgender men, and racial group affiliations of Black, Asian, or Latinx. We further created six gender × race groups (e.g., Black men, Black women, Asian men, …).

We initially followed recommended practices (e.g., [45, 48]) and tried to test the link between the latent classes and the six groups by including it in the model as a categorical auxiliary predictor (dummy coded), or as a known class variable specified to predict the emerging latent class variable. However, due to the relatively small overall sample size, estimation of these models ran into convergence or identification problems, and some emerging cells (e.g., Asian men and Latina in the Disengaged/Non-reactive group) had 5 cases or less (accounting for less than 2% of the sample) which can be considered as negligible statistically [49]. Thus, despite statistical limitations (see Limitation section), similar to Lutz and colleagues [51], we opted to use nonparametric analysis by extracting the latent class membership and conducting a chi-square analysis to examine the likelihood of each of the six identity groups falling into the three latent classes.

The cross table of the six groups and the three latent classes is presented in Table 5. The omnibus chi-square test was significant, χ^2^(10) = 19.37, *p* = .036, effect size = .24. Specifically examining Table 3, Black men appeared to be less likely than expected to fall in the Moderate Engagement group, whereas Latina women appeared to be more likely than expected to be in the Moderate Engagement group. None of the other identity groups showed specific probabilities to fall into any of the latent classes. It is important to note that a power analysis for this Chi-square analysis indicated that with our sample size of 329 non-missing cases and an effect size of .24, the chi-square analysis achieved a power of .87. This suggested that, in contrast to the parametric approach of including the race × gender interactions in the regression mixture model (which was tested but ran into convergence problems), the nonparametric chi-square analysis had adequate power to detect potentially significant relationships.

**Table 5:** Cross-table of race by gender groups and three latent classes.

			Latent Class		
			Triggered/ Reactive	Moderate Engagement	Disengaged/ Non-reactive
	**Black Women**	Count	40	31	21
		SAR	-.3	-1.1	1.9
	**Black Men**	Count	37	17	14
		SAR	1.8	-2.5	1.0
	**Asian Women**	Count	19	20	8
		SAR	-.7	.6	.1
	**Asian Men**	Count	22	15	5
		SAR	1.0	-.4	-.9
	**Latina**	Count	17	29	4
		SAR	-1.7	3.1	-1.8
	**Latino**	Count	13	14	3
		SAR	-.2	1.0	-1.0

*Note*. SAR = Standardized Adjusted Residual. SAR > 2 indicates significantly larger count than expected value, i.e., significantly more likely to fall into this latent class; SAR < -2 indicates significantly smaller count than expected value, i.e., significantly less likely to fall into this latent class.

## Discussion

The current study explored distinct patterns of online social support seeking and anti-racism advocacy in response to online racism, and whether certain patterns were associated with better perceived health and lower alcohol use severity. In line with our general hypothesis, exposure to online racism was significantly associated with greater online social support seeking and anti-racism advocacy engagement in the overall sample, and we found three distinct latent groups of coping patterns: triggered/reactive, moderate engagement, and disengaged/non-reactive groups. Both the triggered/reactive and moderate engagement groups showed similar levels of advocacy engagement [38], but the triggered/reactive group engaged in significantly greater online social support seeking [19]. The disengaged/non-reactive group showed no significant associations with either activity, suggesting that individuals in this group may be detached and passive observers of online racism. Of the three E-RS dimensions, both the triggered/reactive and disengaged/non-reactive groups reported having received significantly more promotion of mistrust messages than the moderate engagement group. The disengaged/non-reactive group reported having received significantly more cultural socialization messages than the moderate group. While no differences in health were observed, alcohol use severity was significantly higher in the triggered/reactive and the disengaged/non-reactive groups compared to the moderate engagement group. Finally, notable differences were observed regarding gender by racial group membership probabilities across the three classes.

One major finding is the contrast between the triggered/reactive and disengaged/non-reactive groups. Both groups reported significantly higher online racism and having received significantly more promotion of mistrust messages than the moderate engagement group, but one group seemed highly triggered while the other appeared disengaged and non-reactive. Considering that those who have been socialized with messages promoting mistrust of others to deal with racial discrimination are more likely to adhere to social mistrust and detachment strategies [27, 30], the disengaged presentation seems fitting in the disengaged/non-reactive group. At an initial glance, individuals in the disengaged/non-reactive group appear that they may perceive online racism experiences to be outside of their coping abilities according to the racial trauma theory [11]. However, the disengaged/non-reactive group also reported receiving significantly more cultural socialization messages than the moderate engagement group, whereas this was not found for the triggered/reactive group. Accordingly, the disengaged/non-reactive group reported a significantly higher baseline online social support seeking and anti-racism advocacy than the triggered/reactive group that seems in line with findings on racial and cultural pride messages being more promotive of racially affirming coping strategies [7]. The higher baseline also suggests that disengagement appears to be salient when it comes to online racism experiences. One possibility is that racial minority adults in the disengaged/non-reactive group may already harbor their own coping strategies or existing support systems that are both self-protective/affirming and mistrustful of others online [31, 32] when they come across racist messages and content on the internet. Thus, they may be less triggered by racist online content, and choose to disconnect from social media and confide in their existing support systems. Additionally, some may employ critical digital media literacy to evaluate and externalize the harmful implications of racist content, messages, and discourses on the internet [52].

On the other hand, the elevated alcohol use severity in the disengaged/non-reactive group suggests some costly implications that are counter to studies that found cultural socialization as a buffer [27] but align with studies that found promotion of mistrust exacerbating alcohol use linked to online and offline racism [12–14, 53]. In particular, the findings provide greater nuance to recent evidence linking online racism to increased alcohol use severity among racial minority emerging adults [12, 13]. One potential explanation may be the nature of racist events experienced online versus offline. It is possible that even for individuals socialized with cultural and racial pride, it may be difficult to sustain these values when bombarded by pervasive and triggering racist content and interactions that invalidate their racial/ethnic being on a persistent basis. Additionally, on the internet, individuals are far more likely to come across groups and polarized racist beliefs (e.g., viral racist posts with many likes, online hate groups) rather than dealing with a particular racist individual and incident in the offline context [3, 4]. In line with the racial trauma Theory [11], such reality may evoke a sense of helplessness, powerlessness, and additive trauma over time [11] that may be harder to manage and feel that it is outside the scope of their coping ability. Even with a strong foundation of cultural and racial pride, individuals in the disengaged/non-reactive group may be overwhelmed with observing and interacting with the pervasive magnitude of polarized racist beliefs on the internet.

Interestingly, despite significant engagement in online social support seeking and anti-racism advocacy, alcohol use severity was similarly high in the triggered/reactive group compared to the disengaged/non-reactive group. These trends seem to suggest an inverse U-shaped relationship where the moderate engagement group may be indicating an optimal level of online social support seeking and advocacy engagement with the lowest risk of alcohol use; beyond which, our anticipation of lower alcohol use severity with greater online social support seeking and advocacy engagement was not the case. Social media use studies have consistently found that active users are likely to retain both benefits and costs impacting their well-being (e.g., [54, 55]). Specifically, given the significantly higher online social support seeking reported by individuals who may be in the triggered/reactive group compared to the moderate group, this trend may be related to the active process of seeking online support. As indicated by the mistrust in online social spaces by racial/ethnic minority individuals [21, 32], perhaps the untold story here is that seeking support in online spaces may come with both benefits and costs of interacting with an online community that may respond either favorably or unfavorably on issues of racism. In fact, some of the interpersonal items of the Perceived Online Racism Scale reflect racial/ethnic minority individuals facing additional aggression due to disagreeing or speaking out against racist comments and posts [2]. Finding a trustworthy online counterspace [19, 20] may not be a straightforward process and come with the burden and dangers of facing additional online racial hate or victimization. Furthermore, given that anti-racism advocacy may be an emotionally charged and exhausting process [33], the greater magnitude of advocacy engagement in this may also reflect the greater psychological burden of being triggered and sustaining resistance. This “side effect” resembles the John Henryism hypothesis that prolonged high-effort coping with chronic psychosocial stressors such as racism may be associated with an elevated risk for negative health outcomes [56, 57]. Ultimately, an alarming trend here is that regardless of whether racial minority individuals approach their coping efforts via active resistance or passive disengagement, both groups seem to be correlated with higher alcohol use severity.

In terms of race by gender group membership probabilities across the three classes, Black men were less likely to fall in the moderate engagement group. Although our chi-square was adequately powered, the adjusted standardized residual value (1.8) for the triggered/reactive group was reaching 2, suggesting that with a greater sample size there may be a significant likelihood for Black men to belong to this group. Particularly for Black men who endorse high levels of traditional masculinity, Black men may engage in more reactive coping strategies that likely align with traditional masculine norms that pressure men to solve their problems actively and independently [57]. These men may report greater alcohol use due to the psychological burden of maintaining highly active support seeking and resistance through advocacy (i.e., John Henryism; [57]). Similarly, although not significant, the residual value (1.9) for the disengaged/non-reactive group was reaching 2 for Black women suggesting that with a greater sample size there may be a significant likelihood for Black women to belong to this group. This is in line with recent studies suggesting that they may use greater disengagement and avoidant strategies to deal with the racism that exacerbates mental health issues [15] and drug use [53]. Interestingly, Latina women were more likely to fall into the moderate engagement group with lower alcohol use severity. Engagement (versus disengagement) coping has been found to have racism coping benefits for Latinx individuals [58] and it appears that there may be culture-specific mechanisms for maintaining a conducive level of online social support seeking and advocacy engagement among these individuals. For Asian Americans, the lack of significant group membership probabilities suggests that their coping and advocacy responses to online racism may be dispersed based on a wide range of individual and contextual factors, some that are more promotive (e.g., family support; [14]) and some that are unhelpful (e.g., colorblind racial attitudes; [3]). Alongside these points, it should also be noted that these group memberships may be reflective of the salience of online racism experiences across identities, particularly for Black women and men. We cannot overlook the fact that the brutal and violent nature of anti-Black racism [17] portrayed online is likely a unique and long-standing traumatic trigger for Black individuals.

### Limitations and future directions

There are several limitations to this study. First, although the overall sample size (*N* = 407) was larger than many existing studies using similar analysis [51], it precluded us from examining the associations between the emerging latent classes and participants’ racial and gender identities using best practices. We acknowledge that saving the latent class membership and conducting additional chi-square analysis as if the class memberships were an observed categorical variable may have important statistical limitations [45, 49]. Therefore, we caution that readers should not overly interpret the inferential statistical tests of racial and gender group differences across the three classes and view the findings as preliminary and exploratory trends that need to be replicated in future studies with a larger sample size. Despite the limitations, the findings suggest important differences that call for future studies with an intersectional lens in assessing online racism and coping strategies across the racial/ethnic minority groups, especially given the crucial distinction between Black women and men’s experiences compared to other racial groups [15].

Relatedly, due to the limited sample size, when examining the race by gender group probabilities of the classes, we were only able to include Black, Asian, and Latinx groups. Other racial/ethnic minority groups (e.g., Native American, Middle East and North African, Multiracial, etc.) were not in the analysis. Despite the statistical rationale, we acknowledge and highlight the limitation of not including specific racial/ethnic minority groups that are the least represented in the population. Future research should specifically focus on these groups and examine potential patterns of their online racism experiences and coping responses.

Third, there are measurement related limitations. We used self-report measures which limit our understanding of our participants’ actual health, alcohol use, and socialization experiences. Additionally, although we noted adequate internal consistency estimates, we asked participants to recall the racial socialization messages they received from their parents in the past which may have affected the validity of their scores. As well, although we provided initial psychometric evidence, the CORS would need to be further validated in future studies. Finally, although most of our measures have been used with good reliability in diverse racial minority samples in prior studies and have measurement invariance evidence (e.g., PORS), some measures (e.g., CORS, ARBI) are relatively new and lack psychometric properties. Thus, our results (particularly the chi-square tests across race by gender groups) should be interpreted with caution and future studies should replicate our findings along with the establishment of psychometric evidence that can help assess adequate group differences in coping approaches.

Lastly, the cross-sectional design of this study does not allow for the test of statistical directionality or causality among the variables. In relating the latent class to racial socialization, alcohol use, and perceived health, we were only able to obtain possible correlational associations and could not make interpretations about directionality or causality. For example, although racial socialization experiences should conceptually precede and impact racial/ethnic minority adults’ coping responses to online racism, with our cross-sectional data we were not able to empirically test this hypothesis. Similarly, we were also not able to ascertain the directionality between the online racism coping classes and the mental health indicators. Additionally, the cross-sectional investigation particularly calls attention to potential confounding variables, such as racism experienced offline and through traditional media (e.g., TV, newspapers) and any racism-related social support received in offline settings. It is likely that our explanation of the different classes needs to be contextualized in additional studies incorporating these factors. For example, our understanding of the disengaged/non-reactive group may evolve if the reason they do not engage in online social support seeking is that they are able to find social support in the offline context. Future researchers may collect longitudinal data or use experimental designs to examine the pathway between racial socialization dimensions, latent classes of responses to online racism, and potential correlates of well-being, while accounting for exposure to racism from multiple avenues (e.g., online and offline).

### Implications for practice

We acknowledge that structural interventions are imperative in addressing online racism and that the burden should not fall on the racial/ethnic minority individuals to find a way to cope or respond to online racial hate. Clearly, policies and organizational changes are much needed to ameliorate the digital burden of online racism on racial/ethnic minority individuals. We hope that our findings inform policymakers and social media platforms to better address online racist speeches and the pervasive racial trauma mediated by these platforms. Importantly, there are several practical implications that may inform individual and group level prevention efforts. First, at the individual level, clinicians working with racial/ethnic minority individuals experiencing online racial violence should consider how their clients have been socialized to deal with racism and their resulting tendency to engage in or disengage from online social support seeking. In particular, clinicians must be aware of the health risks involved with disengagement tendencies and help them begin to consider affirmation strategies to deal with online racism, and eventually promote the development of a network of support and counterspaces to externalize the deficits linked to online racism [28, 53]. Clinicians must also be aware that those who actively engage in online social support seeking and advocacy at high levels may seemingly appear to be dealing with online racism, but they may be engaging in self-medicative alcohol use to cope with the psychological toll of sustaining such process. In such cases, it would be important for clinicians to help clients explore a balanced model of support seeking and advocacy (e.g., the moderate engagement group) that offsets the psychological costs. Additionally, clinicians should work to help these individuals identify trustworthy online/offline counterspaces [7, 20] that can fully aid their support seeking while also recognizing the burden of active coping.

Second, at the family level, socialization messages based on the promotion of mistrust, while they may be helpful in certain situations that call for such mistrust, should be critically re-examined in terms of their usefulness in helping youths and emerging adults deal with online racism. Perhaps it may be helpful to balance the socialization of mistrust and vigilance with messages that reinforce the need for appropriate support seeking [28, 31]. Subsequently, individuals with balanced perspectives may be able to make adaptive decisions on when to take a mistrustful standpoint and when to connect with appropriate support systems to cope with online racism.

Third, at the group level, it seems imperative to develop counterspaces, whether online social media platforms or in relevant institutions such as schools, that validate the negative impact of online racism and seek to maintain a sense of communal support. Although dealing with online racism via detachment and disengagement may be associated with risky alcohol use, it reflects the reality of the difficulty in seeking help and support. For some, the impact of online racism may be severely debilitating that reaching out for support may be stressful and not a simple process. Instead, there should be institutional or organizational initiatives to create counterspaces and communal support systems that are specifically inclusive of and geared for those at the margins of greatest risk. For example, public campaigns focused on anti-racism can contribute to creating a climate that seeks to dismantle racism at multiple levels, including in the online space [59].

## Supporting information

S1 Appendix Coping online with Racism Scale. (DOCX)

## References

[1] Meyer JP, Allen NJ. A three-component conceptualization of organizational commitment. Human resource management review. 1991 Mar 1;1(1):61–89.

[2] Keum BT, Miller MJ. Racism in digital era: Development and initial validation of the Perceived Online Racism Scale (PORS v1. 0). Journal of Counseling Psychology. 2017 Apr;64(3):310–324. doi: 10.1037/cou0000205 28383967

[3] Keum BT, Miller MJ. Racism on the Internet: Conceptualization and recommendations for research. Psychology of violence. 2018 Nov;8(6):782–791.

[4] Tynes BM, Willis HA, Stewart AM, Hamilton MW. Race-related traumatic events online and mental health among adolescents of color. Journal of Adolescent Health. 2019 Sep 1;65(3):371–7. doi: 10.1016/j.jadohealth.2019.03.006 31196779

[5] Stewart A, Schuschke J, Tynes B. Online racism: Adjustment and protective factors among adolescents of color. In Handbook of children and prejudice 2019 (pp. 501–513). Springer, Cham.

[6] Brondolo E, Brady ver Halen N, Pencille M, Beatty D, Contrada RJ. Coping with racism: A selective review of the literature and a theoretical and methodological critique. Journal of behavioral medicine. 2009 Feb;32(1):64–88. doi: 10.1007/s10865-008-9193-0 19127420 PMC3258496

[7] Wang MT, Henry DA, Smith LV, Huguley JP, Guo J. Parental ethnic-racial socialization practices and children of color’s psychosocial and behavioral adjustment: A systematic review and meta-analysis. American Psychologist. 2020 Jan;75(1):1. doi: 10.1037/amp0000464 31058521

[8] Jones SC, Neblett EW. Future directions in research on racism-related stress and racial-ethnic protective factors for Black youth. Journal of Clinical Child & Adolescent Psychology. 2017 Sep 3;46(5):754–66. doi: 10.1080/15374416.2016.1146991 27145002

[9] Smith Lee JR, Robinson MA. “That’s my number one fear in life. It’s the police”: Examining young Black men’s exposures to trauma and loss resulting from police violence and police killings. Journal of Black Psychology. 2019 Apr;45(3):143–84.

[10] Martin-West S. The role of social support as a moderator of housing instability in single mother and two-parent households. Social Work Research. 2019 Mar 1;43(1):31–42.

[11] Carter RT. Racism and psychological and emotional injury: Recognizing and assessing race-based traumatic stress. The Counseling Psychologist. 2007 Jan;35(1):13–05.

[12] Keum BT, Ahn LH. Impact of online racism on psychological distress and alcohol use severity: Testing ethnic-racial socialization and silence about race as moderators. Computers in human behavior. 2021 Jul 1;120:106773.

[13] Keum BT, Cano MÁ. Online racism, psychological distress, and alcohol use among racial minority women and men: A multi-group mediation analysis. American Journal of Orthopsychiatry. 2021;91(4):524. doi: 10.1037/ort0000553 34338543

[14] Wei M, Alvarez AN, Ku TY, Russell DW, Bonett DG. Development and validation of a Coping with Discrimination Scale: factor structure, reliability, and validity. Journal of Counseling Psychology. 2010 Jul;57(3):328. doi: 10.1037/a0019969 21133583

[15] Szymanski DM, Lewis JA. Gendered racism, coping, identity centrality, and African American college women’s psychological distress. Psychology of Women Quarterly. 2016 Jun;40(2):229–43.

[16] Solorzano D, Ceja M, Yosso T. Critical race theory, racial microaggressions, and campus racial climate: The experiences of African American college students. Journal of Negro education. 2000 Jan 1:60–73.

[17] Jones SC, Anderson RE, Gaskin-Wasson AL, Sawyer BA, Applewhite K, Metzger IW. From “crib to coffin”: Navigating coping from racism-related stress throughout the lifespan of Black Americans. American Journal of Orthopsychiatry. 2020;90(2):267. doi: 10.1037/ort0000430 32105125 PMC8807348

[18] Keum BT. Qualitative examination on the influences of the internet on racism and its online manifestation. International Journal of Cyber Behavior, Psychology and Learning (IJCBPL). 2017 Jul 1;7(3):13–22.

[19] Eschmann R. Digital resistance: How online communication facilitates responses to racial microaggressions. Sociology of Race and Ethnicity. 2021 Apr;7(2):264–77.

[20] George Mwangi CA, Bettencourt GM, Malaney VK. Collegians creating (counter) space online: A critical discourse analysis of the I, Too, Am social media movement. Journal of Diversity in Higher Education. 2018 Jun;11(2):146.

[21] To A, Sweeney W, Hammer J, Kaufman G. " They Just Don’t Get It": Towards Social Technologies for Coping with Interpersonal Racism. Proceedings of the ACM on Human-Computer Interaction. 2020 May 28;4(CSCW1):1–29.

[22] Bowman PJ, Howard C. Race-related socialization, motivation, and academic achievement: A study of Black youths in three-generation families. Journal of the American Academy of Child Psychiatry. 1985 Mar 1;24(2):134–41. doi: 10.1016/s0002-7138(09)60438-6 3989153

[23] Elder GH Jr. Time, human agency, and social change: Perspectives on the life course. Social psychology quarterly. 1994 Mar 1:4–15.

[24] Bandura A. Self-efficacy: toward a unifying theory of behavioral change. Psychological review. 1977 Mar 1;84(2). doi: 10.1037//0033-295x.84.2.191 847061

[25] Lesane-Brown CL. A review of race socialization within Black families. Developmental Review. 2006 Dec 1;26(4):400–26.

[26] Hughes D, Rodriguez J, Smith EP, Johnson DJ, Stevenson HC, Spicer P. Parents’ ethnic-racial socialization practices: a review of research and directions for future study. Developmental psychology. 2006 Sep;42(5):747. doi: 10.1037/0012-1649.42.5.747 16953684

[27] Grindal M. Ethnic–racial socialization, social bonds, and college student substance use. Deviant behavior. 2017 Oct 3;38(10):1102–19.

[28] Hope EC, Volpe VV, Briggs AS, Benson GP. Anti‐racism activism among Black adolescents and emerging adults: Understanding the roles of racism and anticipatory racism‐related stress. Child Development. 2022 Feb 25. doi: 10.1111/cdev.13744 35211959 PMC9306571

[29] Thai CJ, Lyons HZ, Lee MR, Iwasaki M. Microaggressions and self-esteem in emerging Asian American adults: The moderating role of racial socialization. Asian American Journal of Psychology. 2017 Jun;8(2):83.

[30] Chávez NR, French SE. Ethnicity‐related stressors and mental health in Latino Americans: The moderating role of parental racial socialization. Journal of Applied Social Psychology. 2007 Sep;37(9):1974–98.

[31] Su J, Kuo SI, Derlan CL, Hagiwara N, Guy MC, Dick DM. Racial discrimination and alcohol problems among African American young adults: Examining the moderating effects of racial socialization by parents and friends. Cultural diversity and ethnic minority psychology. 2020 Apr;26(2):260. doi: 10.1037/cdp0000294 31328948 PMC6980251

[32] Ortiz SM. “You can say I got desensitized to it”: How men of color cope with everyday racism in online gaming. Sociological Perspectives. 2019 Aug;62(4):572–88.

[33] Singh AA, Hofsess CD, Boyer EM, Kwong A, Lau AS, McLain M, et al. Social justice and counseling psychology: Listening to the voices of doctoral trainees. The Counseling Psychologist. 2010 Aug;38(6):766–95.

[34] Saunders JB, Aasland OG, Babor TF, De La Fuente JR, Grant M. Development of the alcohol use disorders identification test (AUDIT): WHO collaborative project on early detection of persons with harmful alcohol consumption‐II. Addiction. 1993 Jun;88(6):791–804. doi: 10.1111/j.1360-0443.1993.tb02093.x 8329970

[35] Reinert DF, Allen JP. The alcohol use disorders identification test (AUDIT): a review of recent research. Alcoholism: Clinical and Experimental Research. 2002 Feb;26(2):272–9. 11964568

[36] Erford BT, Sriken J, Sherman MF, Hibbs JS, Smith HL, Kipper-Smith A, et al. Psychometric analysis, internal structure, and measurement invariance of the Alcohol Use Disorders Identification Test (AUDIT) scores from a large university sample. Measurement and Evaluation in Counseling and Development. 2021 Jul 3;54(3):188–205.

[37] Hughes D, Johnson D. Correlates in children’s experiences of parents’ racial socialization behaviors. Journal of Marriage and family. 2001 Nov;63(4):981–95.

[38] Pieterse AL, Utsey SO, Miller MJ. Development and initial validation of the anti-racism behavioral inventory (ARBI). Counselling Psychology Quarterly. 2016 Oct 1;29(4):356–81.

[39] Davis CH, Krafft J, Hicks ET, Levin ME. The role of psychological inflexibility and perspective taking in anti-racism and anti-sexism. Personality and individual differences. 2021 Jun 1;175:110724.

[40] Hays RD, Spritzer KL, Thompson WW, Cella D. US general population estimate for “excellent” to “poor” self-rated health item. Journal of general internal medicine. 2015 Oct;30(10):1511–6. 25832617 10.1007/s11606-015-3290-x PMC4579204

[41] Carver CS. You want to measure coping but your protocol’too long: Consider the brief cope. International journal of behavioral medicine. 1997 Mar;4(1):92–100. 16250744 10.1207/s15327558ijbm0401_6

[42] van Ingen E, Wright KB. Predictors of mobilizing online coping versus offline coping resources after negative life events. Computers in Human Behavior. 2016 Jun 1;59:431–9.

[43] Kline RB. Principles and practice of structural equation modeling. Guilford publications; 2015 Nov 3.

[44] Hu LT, Bentler PM. Cutoff criteria for fit indexes in covariance structure analysis: Conventional criteria versus new alternatives. Structural equation modeling: a multidisciplinary journal. 1999 Jan 1;6(1):1–55.

[45] Asparouhov T, Muthén BO. Appendices for auxiliary variables in mixture modeling: 3-step approaches using Mplus manually versus all steps done automatically. Structural Equation Modeling. 2014.

[46] Muthén BO. Latent variable mixture modeling. InNew developments and techniques in structural equation modeling 2001 Mar 1 (pp. 21–54). Psychology Press.

[47] Vaughan EL, Wong YJ, Middendorf KG. Gender roles and binge drinking among Latino emerging adults: a latent class regression analysis. Psychology of Addictive Behaviors. 2014 Sep;28(3):719. doi: 10.1037/a0037406 25222172

[48] Muthén LK, Muthén BO. Mplus (Version 8)[Computer software]. Los Angeles, CA: Author.

[49] Lanza ST, Tan X, Bray BC. Latent class analysis with distal outcomes: A flexible model-based approach. Structural equation modeling: a multidisciplinary journal. 2013 Jan 1;20(1):1–26. doi: 10.1080/10705511.2013.742377 25419096 PMC4240499

[50] Nylund KL, Asparouhov T, Muthén BO. Deciding on the number of classes in latent class analysis and growth mixture modeling: A Monte Carlo simulation study. Structural equation modeling: A multidisciplinary Journal. 2007 Oct 23;14(4):535–69.

[51] Lutz W, Prinz JN, Schwartz B, Paulick J, Schoenherr D, Deisenhofer AK, et al. Patterns of early change in interpersonal problems and their relationship to nonverbal synchrony and multidimensional outcome. Journal of Counseling Psychology. 2020 Jul;67(4):449. doi: 10.1037/cou0000376 32614226

[52] Yosso TJ. Critical race media literacy: Challenging deficit discourse about Chicanas/os. Journal of popular film and television. 2002 Jan 1;30(1):52–62.

[53] Stevens-Watkins D, Perry B, Harp KL, Oser CB. Racism and illicit drug use among African American women: The protective effects of ethnic identity, affirmation, and behavior. Journal of Black Psychology. 2012 Nov;38(4):471–96. doi: 10.1177/0095798412438395 24482547 PMC3904443

[54] Bányai F, Zsila Á, Király O, Maraz A, Elekes Z, Griffiths MD, et al. Problematic social media use: Results from a large-scale nationally representative adolescent sample. PloS one. 2017 Jan 9;12(1):e0169839. doi: 10.1371/journal.pone.0169839 28068404 PMC5222338

[55] Keum BT, Wang YW, Abebe I, Callaway J, Cruz T, O’Connor S. Benefits and Harms of Social Media Use: A Latent Profile Analysis of Emerging Adults. Curr Psycho. Forthcoming 2022. doi: 10.1007/s12144-022-03473-5 35891891 PMC9302950

[56] Bennett GG, Merritt MM, Sollers Iii JJ, Edwards CL, Whitfield KE, Brandon DT, et al. Stress, coping, and health outcomes among African-Americans: A review of the John Henryism hypothesis. Psychology & Health. 2004 Jun 1;19(3):369–83.

[57] Matthews DD, Hammond WP, Nuru-Jeter A, Cole-Lewis Y, Melvin T. Racial discrimination and depressive symptoms among African-American men: The mediating and moderating roles of masculine self-reliance and John Henryism. Psychology of Men & Masculinity. 2013 Jan;14(1):35. doi: 10.1037/a0028436 30364828 PMC6197817

[58] Sanchez D, Adams WN, Arango SC, Flannigan AE. Racial-ethnic microaggressions, coping strategies, and mental health in Asian American and Latinx American college students: A mediation model. Journal of counseling psychology. 2018 Mar;65(2):214. doi: 10.1037/cou0000249 29543476 PMC10371219

[59] Birk M. Do You Hear Me? A Critical Review of the Voice of Racism Anti-racism Education Campaign from Aotearoa New Zealand. New Zealand Journal of Educational Studies. 2022 Jan;1–16. 10.1007/s40841-022-00239-2 PMC8780042 37521819

