# Supplementary material for: Coping with online racism: Patterns of online social support seeking and anti-racism advocacy associated with online racism, and correlates of ethnic-racial socialization, perceived health, and alcohol use severity
Source: PLoS One. 2022 Dec 2;17(12):e0278763. doi: 10.1371/journal.pone.0278763 (PMC9718414; doi:10.1371/journal.pone.0278763)
Supplement: S1 Appendix — (DOCX) [file pone.0278763.s001.docx]

**S1 Appendix. Coping Online with Racism Scale.**

**Coping Online with Racism Scale**

Please rate the extent to which you engage in the following activities in response to online racism on the internet (e.g., encountering racist trolls; receiving racist comments; seeing racist posts, photos, or videos; encountering viral media on racial violence toward racial/ethnic minority individuals; encountering online hate groups, etc.).

1-*never,* 2-*rarely,* 3-*sometimes,* 4-*very often,* 5-*very often*

1. I let out my frustration or anger in online groups/social media.
2. I vent about my experiences in online groups/social media.
3. I seek social support in online groups/social media.
4. I seek emotional support in online groups/social media.
5. I look for online resources.
6. I watch online videos that share my frustration or anger.
7. I read online articles that share my frustration or anger.
8. I follow or connect with people who share my experiences.
9. I follow or connect with people who helps me to cope.
10. I block or unfollow people who do not share my goal on abolishing racism.
